# Reduced effects of social feedback on learning in Turner syndrome

**DOI:** 10.1038/s41598-023-42628-7

**Published:** 2023-09-22

**Authors:** Hanna Björlin Avdic, Claes Strannegård, Hedvig Engberg, Charlotte Willfors, Ida Nordgren, Louise Frisén, Angelica Lindén Hirschberg, Mona Guath, Ann Nordgren, Johan Lundin Kleberg

**Affiliations:** 1 Centre for Psychiatry Research, Department of Clinical Neuroscience, Karolinska Institutet & Stockholm Health Care Services, Region Stockholm, Stockholm, Sweden; 2 Department of Molecular Medicine and Surgery, Center for Molecular Medicine, Karolinska Institutet, Stockholm, Sweden; 3 Division of Cognition and Communication, Department of Applied IT, University of Gothenburg, Gothenburg, Sweden; 4 Department of Women’s and Children’s Health, Karolinska Institutet & Department of Gynaecology and Reproductive Medicine, Karolinska University Hospital, Stockholm, Sweden; 5 Department of Psychology, Uppsala University, Uppsala, Sweden; 6 Department of Laboratory Medicine, Institute of Biomedicine, University of Gothenburg, Gothenburg, Sweden; 7 Department of Clinical Genetics and Genomics, Sahlgrenska University Hospital, Gothenburg, Sweden; 8 Department of Clinical Genetics, Karolinska University Hospital, Stockholm, Sweden; 9 Department of Psychology, Stockholm University, Stockholm, Sweden

**Keywords:** Cognitive neuroscience, Learning and memory, Human behaviour, Autism spectrum disorders

## Abstract

Turner syndrome is a genetic condition caused by a complete or partial loss of one of the X chromosomes. Previous studies indicate that Turner syndrome is associated with challenges in social skills, but the underlying mechanisms remain largely unexplored. A possible mechanism is a reduced social influence on learning. The current study examined the impact of social and non-social feedback on learning in women with Turner syndrome (n = 35) and a sex- and age-matched control group (n = 37). Participants were instructed to earn points by repeatedly choosing between two stimuli with unequal probabilities of resulting in a reward. Mastering the task therefore required participants to learn through feedback which of the two stimuli was more likely to be rewarded. Data were analyzed using computational modeling and analyses of choice behavior. Social feedback led to a more explorative choice behavior in the control group, resulting in reduced learning compared to non-social feedback. No effects of social feedback on learning were found in Turner syndrome. The current study thus indicates that women with Turner syndrome may be less sensitive to social influences on reinforcement learning, than the general population.

## Introduction

Turner syndrome is a genetic condition in females caused by a complete or partial absence of the second sex chromosome. This sex chromosome aneuploidy has a reported incidence of 1:1700–1:2000 female births^1,2^ and a wide-spread of manifestations as well as a wide phenotypic penetrance^3,4^. Gonadal dysgenesis and ovarian failure leading to infertility are common in Turner syndrome as well as short stature, cardiovascular-, endocrinological- and gastrointestinal diseases^5^. The cognitive profile of Turner syndrome can vary widely, still it is often described with an overall intellectual ability within the normal range but with an uneven profile characterized by relative strengths in verbal functions compared to visuo-spatial abilities^6–8^. Other impairments commonly associated with Turner syndrome include impaired executive functions, such as working memory, cognitive flexibility, and attention^9–11^.

Previous studies have reported that individuals with Turner syndrome often experience difficulties in social relationships with peers and have fewer partners^12–15^. A phenotypic overlap with autism spectrum disorder (ASD) has been suggested, but the nature of this overlap remains insufficiently understood^15^. Challenges in social withdrawal are described, however, often with an accompanying desire for social interaction^13,16–19^. To further understand the psychosocial phenotype in Turner syndrome, as pointed out in a recent review by Wolstencroft & Skuse 2019, there is an urgent need for objective methods to assess and measure social difficulties and social skills^13^. However, the definition of social skills is broad, and the assessment methods differ, making it difficult to understand and compare results between studies and over time. Another complicating factor is that assessment methods for examining social skills often rely on self-report. Individuals with Turner syndrome tend to overestimate their social skills and self-report fewer social difficulties than reported by their parents or others^15,18,20^.

Difficulties in processing facial expressed emotions is suggested as a contributing factor in social interaction challenges. Turner syndrome has been associated with difficulties in recognition of negative emotions, most consistently for faces displaying fear^21–23^, sadness or disgust^24^. Furthermore, when processing faces displaying positive emotions such as joy, changes in brain activation in several regions associated with social cognition and attention have been described in individuals with Turner syndrome^23^.

Faces and facial expressions in others function as powerful reinforcers of behavior^25^. These effects are further modulated by the mental states we attribute to observed others^26^. The influence of faces on learning extend beyond specific emotions, as the mere presence of others can affect learning^27^. Typically, people tend to rely more on facial cues for learning when other forms of feedback is unreliable or when the task to learn is characterized by a high degree of uncertainty^28,29^. Importantly, reliance on facial expressions for learning will only be adaptive when the information available through social cues is more reliable than what can be achieved through other sources of feedback^29^. However, despite multiple reports of atypical face perception in Turner syndrome, no study has so far examined how learning is affected by facial expressions in women with the condition^24,30^.

The relationship between actions and outcomes can be *deterministic,* meaning that the same action leads to the same result in every case. More commonly, we need to learn *probabilistic* associations between actions and outcomes where humans and animals typically rely on a form of learning known as *reinforcement learning *^29,31^. In reinforcement learning, associations between actions and expected resulting outcomes are gradually updated through *prediction errors*, or the mismatch between expectation and outcome^31,32^. In a hypothetical situation, a child may learn that going to the playground is likely to lead to the opportunity to play with friends. However, this positive outcome will not follow on every single case (for example, the friends may be away on holidays). Therefore, the child will have to learn that going to the playground maximizes the probability of a favorable outcome, but also understand that there is an inherent uncertainty to this rule.

To successfully learn how to navigate a probabilistic environment, the individual must balance between relying on their learned strategy (exploit), and to explore the environment further by occasionally deviating from this strategy. This is known as the *exploration–exploitation* balance^33^. Reinforcement learning is dependent on a well-described neural circuits including the striatum and the meso-cortical dopaminergic tracts^29^, and recent research suggest that executive functions may interact with this network, and thereby modulate reinforcement learning^34^.

Social feedback (such as facial expressions of emotion in others) strongly affects, and typically improves reinforcement learning^25,35–37^. However, direct comparisons with other types of feedback such as money or symbolic rewards shows that the later are often even more efficient as reinforcers in probabilistic learning tasks. For example, participants were slower to learn with social as compared to non-social feedback in a study by Lin et al. (2012), despite activation of overlapping brain mechanisms between the two types of feedback^37^. The exploration–exploitation balance typically shifts towards exploration in socially rich environments^33^, and theoretical models^38^ indicate that this shift is adaptive at the individual level.

Social reinforcement learning is currently studied in a wide range of neurodevelopmental conditions^25,31,32^, and alterations have been found in conditions such as ASD^39,40^, depression^41–43^, and genetic conditions associated with altered social interaction^44^. No studies have been conducted in Turner syndrome. However, there are many reasons to suspect altered social reinforcement learning in this group. First, as described above, challenges with social interaction are common, although the nature of these difficulties remain insufficiently understood^6,13^. Secondly, magnetic resonance imaging (MRI) of the brain in participants with Turner syndrome has shown a neuroanatomical phenotype with structural alterations of brain regions involved in both reinforcement learning and social processing^10,23,45,46^. Thirdly, studies using eye tracking have documented atypical visual attention to emotional faces, which could be linked to a reduced ability to interpret and/or learn from facial cues^21,22^. Thus, in this study, we hypothesized that women with Turner syndrome would have a reduced effect of social (as compared to non-social) feedback on reinforcement learning.

In addition to traditional behavioral indices of choice behavior and learning, we analyzed data using computational modeling methods. In computational modeling, mathematical models are fitted to the data in order to extract latent variables representing underlying cognitive processes^47^ which, for example, has been instrumental in understanding the neural basis of reinforcement learning^48,49^. Computational modelling methods are also increasingly used to characterize phenotypes and examine the potential underlying mechanisms of psychiatric symptoms^31,43,44,50,51^. Furthermore, parameters from computational modeling of reinforcement learning data have been hypothesized as a promising biomarker for mental health conditions affecting reward-driven behavior in theoretical frameworks such as the The National Institute of Mental Health's Research Domain Criteria (RDoC) Initiative^52^.

To the best of our knowledge, this study was the first to examine reinforcement learning in Turner syndrome. If social reinforcement alterations are seen in women with Turner syndrome, these could potentially constitute a link in the developmental pathway from genotype to social behavioral atypicalities. If so, the alterations could be feasible as a means of evaluating treatments and interventions essential for early detection and individualized care.

## Methods

### Participants

Participants with Turner syndrome (final *n* = *35*) were compared to a control group of adult women without known genetic disorders (*final n* = *37*).

Individuals with Turner syndrome were recruited at the Karolinska University Hospital and by advertising through a patient organization. Inclusion criteria was a diagnosis of Turner syndrome, age > 15 years and fluency in Swedish. Initially, 38 participants agreed to participate and completed the task. Of these, three were excluded due to low data quality. Of the 35 participants, 26 had completed the Vocabulary subtest of cognitive ability in the Wechsler Intelligence Scale for Adults, 4th Edition (WAIS-IV)^53^ which was included as a screening measure for cognitive ability. In 30 of the participants with Turner syndrome, the diagnosis was confirmed through medical records. The other five participants reported that they had received a diagnosis of Turner syndrome in general health care but did not provide medical records. Seven participants with Turner syndrome completed one condition only (social: *n* = *3, non-social: n* = *4*).

The control group was recruited through online advertisement at the Karolinska Insitutet web page. Inclusion criteria were female gender and age > 15, no diagnose of Turner syndrome and fluency in Swedish. Exclusion criteria were ongoing medication with known psychotropic effects, any psychiatric or neurological disorder, or suspected genetic condition. Initially, 47 women expressed interest to participate and 40 of them completed the task. Also in this group, three participants were excluded due to low data quality, by not exploring both options for the task, or by showing a pattern of responses indicating inattention. Of the final group, 18 completed the Vocabulary subtest in WAIS-IV as a screening for cognitive ability^53^. As can be seen in Table 1, no significant differences in age or Vocabulary scores were found between the Turner syndrome and control group.

**Table 1:** Demographic characteristics.

Measure	Turner syndrome	Control	Group comparison ‡		
	M(SD)	M(SD)	*t*	*p*	*d*
Age	33.83 (10.80)	38.10 (15.05)	1.64	0.105	0.39
WAIS Vocabulary (scaled score)+	9.00 (2.83)	10.00 (1.65)	1.35	0.185	0.41

^+^Based on n = 26 in the Turner syndrome group and n = 18 in the control group.

^‡^Independent Samples t-test.

t = t-statistic; d = Cohen’s d.

The Swedish Ethical Review Authority approved the study, which followed the tenets of the Declaration of Helsinki. Written informed consent was obtained from all participants. The control group was recruited in the context of a larger study which also includes children with intellectual disability and other rare genetic disorders linked to intellectual disability as well as typically developing males. The control group is therefore partly overlapping with the group reported in Kleberg et al. (2023)^44^. Participants who did not explore both options of the task (> 90% choices of one stimulus), (control: *n* = 2, Turner syndrome: *n* = 2) were excluded.

### Task and procedure

**Figure 1:**
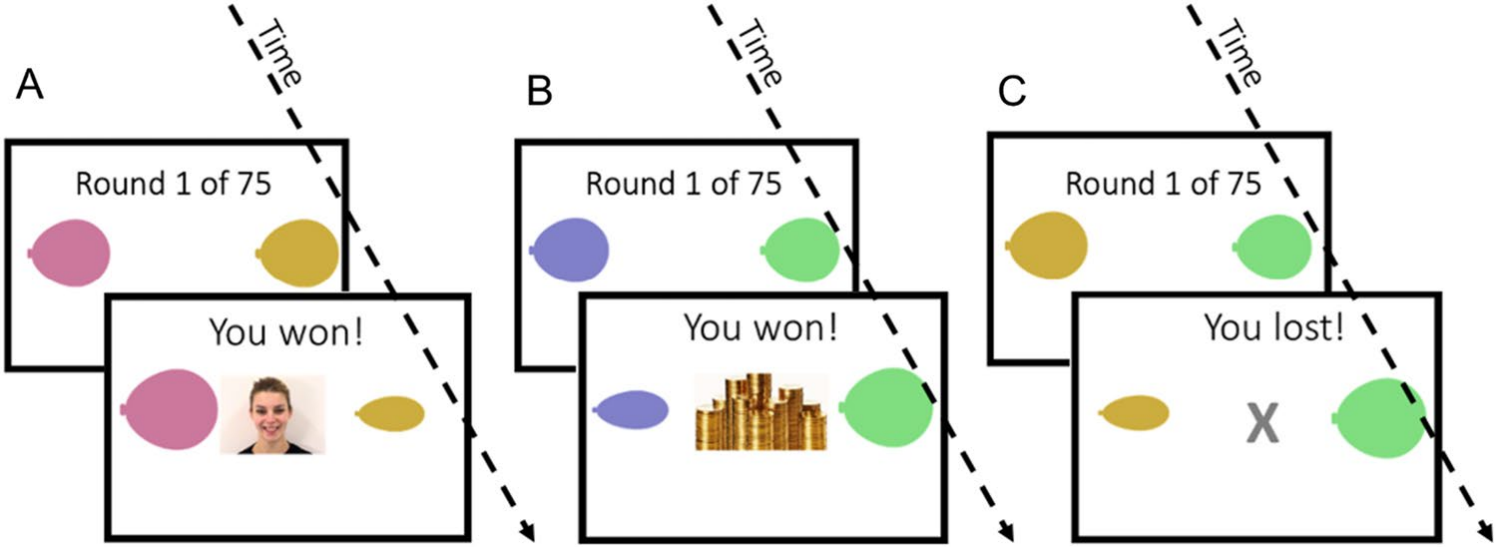
Experimental design. In counterbalanced order, participants completed 75 trials in each condition rounds. Choosing the correct option was followed by receiving (A) social feedback (animation of a smiling face, social condition) or(B) non-social feedback (animation of a stack of gold coins, non-social feedback). Incorrect choices, in both condition rounds (**A** and **B**), were followed by an animation of the letter “X” moving towards the participant, (**C**). In both conditions, the reward probabilities of the stimuli were 2/3 and 1/3 respectively. Stimulus color and position of the better stimulus (left/right) were counterbalanced between participants and conditions.

The reinforcement learning task (described in Fig. 1) was adapted from Kleberg et al. (2023)^44^. Participants completed two versions of the tasks (henceforth the social and non-social conditions) using either a tablet or computer. Both versions included 75 trials of a choice between two stimuli with a probability of win a point of 2/3 and 1/3 respectively. Thus, a hypothetical participant who consistently chose the correct stimulus in all 75 trials would win a point in two thirds of those cases. However, this most rewarding choice in the long run is hereafter referred to as the *correct choice* for the sake of simplicity*.* Mastering the task therefore requires the participant to learn through exploration which action is most likely to result in a favorable outcome.

The following instructions were given on the screen (in Swedish):

“You will play a game. Your task is to try to inflate balloons. You will see two balloons on the screen and choose one of them. If you succeed, you will see that the balloon is inflated. You will choose multiple times. One of the two balloons is better. You will have to explore to find out which of them it is”.

Following each choice, participants received feedback on whether their choice resulted in a win or a loss (note that, as described above, due to the probabilistic nature of the task, a correct choice did not always result in winning a point and an incorrect choice not always in a loss). Feedback for winning a point was a written message on the screen (“you won!”) and in the social condition an animation of a smiling woman, while in the non-social condition a pile of gold coins. Feedback for losses was always an animation of the letter “X” moving towards the participant along with the text “you lost!” Participants completed both conditions which were presented in one of two potential task orders (social–non-social, henceforth *S–N*, or non-social–social, henceforth *N–S*). Task order was counterbalanced between participants and groups so that every second participant in each group was assigned to the S–N task order and every second to the N–S task order.

Directly following each condition, participants were asked to rate their affective experience of winning a point, losing a point, and receiving social or non-social feedback on a seven-point ascending Likert scale with the following anchors: *1* = *Very unpleasant, 4* = *Neither pleasant or unpleasant, and 7* = *Very pleasant.*

Participants performed the experimental task from home via the Internet using a computer or tablet, with the exception of two participants who performed the task from a computer at a research facility (control, n = 2). The task was implemented in Pavlovia which enables stimulus presentation and reaction time measurement with millisecond precision^54^. Participants were instructed to complete the task by themselves in an environment where they would not be disturbed.

### Computational modeling

The fit of several reinforcement learning models and alternative models were compared using the Akaike information criterion (AIC). The winning models were further validated through simulation (see *Supplementary materials* for a full description).

Validation analyzes based on the AIC showed that, compared with alternative models, the delta rule (also known as the Rescorla-Wagner reinforcement learning model), provided a better fit to the data in the social condition in the Turner syndrome group, and in both social- and non-social conditions in the control group. In the social condition in the Turner syndrome group a slightly better fit was found in an alternative reinforcement learning model (see *Supplementary materials*) but to facilitate comparison, the delta rule (Rescorla-Wagner model) was used in both groups. Simulation-based validation analyses demonstrated adequate parameter recovery. All reinforcement models had a better fit to the data than a control model assuming random choices, which had relatively poor fit, suggesting that participants were actively engaged in the task (Supplementary materials, Figure S1).

The Rescorla–Wagner reinforcement learning model updates the expected value of the choice *c* at trial *t* after observing the reward *r* according to the delta rule:

1 $$V{(c)}_{t+1}={V(c)}_{\begin{array}{c}t\\ \end{array}}+ \alpha ({r}_{t}-{V\left(c\right)}_{t})$$

Here, the term $({r}_{t}-{V\left(c\right)}_{t})$ the *prediction error*, or the difference between the expected and the received reward. The prediction error is negative if the outcome was less than expected and positive if better than expected. The learning rate α ranges from 0 to 1 and governs the degree to which the prediction error is taken into account. At α = 1, V(c) is fully updated at each trial. If α = 0, no learning takes place. Note that *learning rate* here simply refers to the degree of updating, and that a higher learning rate does not necessarily indicate more efficient learning. In Eq. (1), only the value of the chosen option *c* is updated, whereas the unchosen option is not updated after receiving feedback. This model is henceforth referred to as the *single update model*. Here, *r* was defined as 1 if the outcome was a win, and as − 1 if the outcome was a loss. We set *V*(*c*) to 0 at the first trial, to reflect that participants were ignorant about reward probabilities at the onset of the experiment. Expected values are transformed into choice probabilities by using the SoftMax function:

2 $$P({c}_{t})=\frac{\mathrm{exp}(\beta V\left({c}_{t}\right))}{\mathrm{exp}(\beta V\left({c}_{t}\right))+\mathrm{exp}(\beta V\left({nc}_{t}\right))}$$

**Figure 2:**
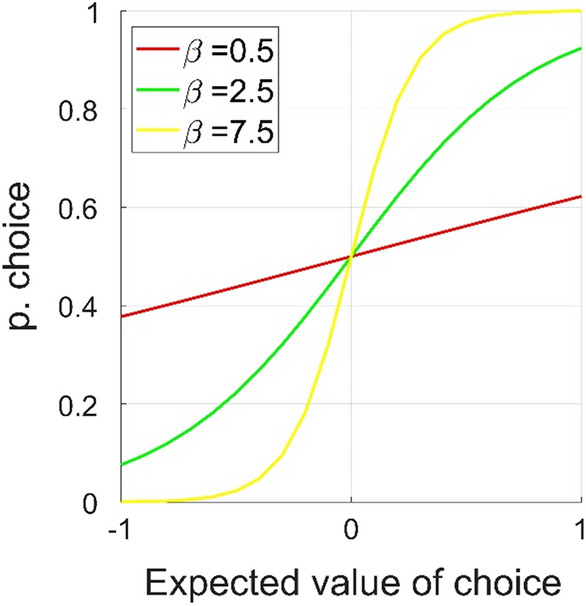
Exploitation-exploration balance. Effects of different values of the reinforcement learning parameter β (exploitation-exploration balance). Higher values of β leads to more deterministic choices, so that a stimulus with higher expected value is chosen with higher probability. Lower levels of β leads to a higher probability of explorative choices.

Here, P(c_t_) represents the probability of making choice *c* at trial *t. V*(*c*_*t*_) is the expected value of choice *c* and *V*(*nc*_*t*_) the value of the non-chosen stimulus, and *exp* denotes the exponential function with the base *e*. The parameter β ranging from 0 to infinity governs the rate of exploration vs. exploitation, with higher values indicating more deterministic choices (i.e., a higher probability of making the choice with the higher expected value). The effect of *β* on choice probability is illustrated in Fig. 2. The standard reinforcement model therefore has two free parameters, α and β. Note that only the expected value of the chosen action is updated in the standard reinforcement learning (Eq. 1) whereas the unchosen option is not updated after receiving feedback.

### Dependent variables

In a preliminary analysis, we compared self-ratings of affective experiences of winning or losing points and of receiving social or non-social feedback following wins as well as reaction times and reaction time variability (individual medians and median absolute distances (MADs)). The following dependent variables representing directly observable choice behavior were analyzed: the proportion of trials where the participant chose the balloon which was more likely to get a winning point (*%correct*), the proportion of trials where the participants switched from the previously chosen balloon (*%volatility*), the proportion of switches following wins (*%switch-win*) and losses *(%switch-lose*). Choice behavior data were grouped into five blocks of 15 trials each. While %correct reflects task performance, the choice behavior variables provide insights into strategy and cognitive processes involved in solving the task. Since reinforcement learning tasks require a balance between exploration and exploitation of previously acquired knowledge, neither high nor low values on these variables are adaptive in every context. For example, a high %volatility early on in an unknown task may be adaptive since it leads the agent to explore and learn about the underlying reward probabilities associated with the choices. In contrast, a low %volatility is likely to be adaptive during later stages of the task when the agent has learned about the reward probability of the task and has less need for exploration^49^. High volatility during later stages of the task may instead reflect inattention. From the computational modeling analysis, the parameters α and β were analyzed.

### Statistical analyses

Data visualizations indicated skewed distributions of all dependent variables. After square root transformation, choice behavior and reaction time variables were approximately normally distributed. Choice behavior data were analyzed using linear mixed effects models (LMMs) with condition (social, non-social), block (1–5) and task order (1 or 2) as within-subjects factors and group (control, Turner syndrome) as between-subjects factor. For ease of interpretation, untransformed parameter values are reported. Interaction effects between group and condition were added to test the hypothesis that social feedback would have a different effect in the Turner syndrome group and controls while additional interaction terms between group and block and group and task order were included to account for potential group differences in the development of choice behavior over time. Due to the wide age range, age was added as a covariate. Random intercept for individual was included to account for repeated measures.

β values (one β value for each condition per participant) were normally distributed after square root transformation and were analyzed using an LMM with main effects of group, condition, and task order and two-way interactions between group and condition and group and task order. Age was added as a covariate.

Significant interaction effects found in LMMs were followed up using Bonferroni-corrected pairwise contrasts using the emmeans package in R^55^. p-values were calculated using the Kenward-Roger method. α values deviated from a normal distribution after square root transformation, and these data were therefore analyzed using non-parametric Wilcoxon tests. The threshold for statistical significance was set to *p* = 0.05. All analyses were conducted using the lme4^56^, lmerTest^57^ and emmeans packages in R^55^.

### Power analysis

A simulation-based power analysis conducted using the *simr* package in R^58^ based on the observed random effects structure in Kleberg et al. (2023) indicated that the study had > 80% power to detect within-group effects of condition corresponding to a 7% difference in the proportion of correct responses, which was considered a meaningful effect^44^.

## Results

### Reaction time

A significant main effect of block was found, reflecting quicker reaction times during later trials [b =  − 0.04, se = 0.02, *t* = − 2.94, *p* = 0.003]. There was also a significant effect of condition, reflecting slower reaction times in the social than the non-social condition [b = 0.15, se = 0.07, t = 2.85, *p* = 0.005], but no effect of group [b = 0.31, se = 0.18, t = 1.71, *p* = 0.091]. No significant interaction effects were found for group x condition [b = 0.03, se = 0.06, t = 0.72, *p* = 0.471], group x block [b = − 0.05, se = 0.02, t = − 1.64, *p* = 0.101], group x task order [b = 0.38, se = 0.25, t = 1.40, *p* = 0.17], or condition x block [b = − 0.03, se = 0.02, t = 1.97, *p* = 0.05]. To sum up, these results indicate that participants’ responses became quicker during later trials within each round. Participants were also quicker to respond in the non-social than in the social condition. No evidence was found that these effects interacted with group status (Turner syndrome, control).

### Reaction time variability

A significant main effect of condition was found, reflecting higher reaction time variability in the social as compared to the non-social condition [b = 0.19, se = 0.05, t = 3.81, *p* < 0.001]. The main effects of group [Turner syndrome vs. Control: b = 0.19, se = 0.07, t = 1.94, *p* = 0.054] and block [b = − 0.01, se = 0.01, *p* = 0.56] and task order [b = 0.08, se = 0.06, t = 1.27, *p* = 0.21] were not significant. No significant interaction effects were found between group and condition, group and block, or group and task order (all *p* > 0.33). However, the condition by block interaction was significant [b = 0.04, se = 0.02, t = 2.75, *p* = 0.006]. Bonferroni-corrected follow up-tests showed that reaction time variability decreased significantly over the course of the task in both the social [b = − 0.39, se = 0.02, t = 16.82, *p* < 0.001] and the non-social conditions [b = − 0.30, se = 0.02, t = 13.35, *p* < 0.001], although with a larger effect in the former. To sum up, these results suggest that reaction time variability was larger in the social than in the non-social condition, and that variability decreased over time within the experiment. No evidence was found that reaction time variability varied with group status.

### Ratings of affective experiences

Affective experience ratings are shown in Table 2*.* As described under *Methods,* a rating of 4 indicated a neutral affective response. Therefore, we tested whether the ratings differed from 4 using uncorrected one-sample t-tests. Ratings of winning a point were significantly higher than 4 (Turner syndrome, social condition: t = 3.61, *p* = 0.001; Turner syndrome, non-social condition: t = 3.48, *p* = 0.002; Control group, social condition: t = 6.09, *p* < 0.001; Control group, non-social condition: t = 8.30, *p* < 0.0001), indicating that winning a point was experienced as positive. Conversely, ratings of losing a point were significantly lower than 4, indicating negative affect (Turner syndrome, social condition: t = − 2.54, *p* = 0.01; Turner syndrome, non-social condition: t = − 3.74, *p* < 0.001; Control group, social condition: t = − 6.29, *p* < 0.0001; Control group, non-social condition: t = − 5.73, *p* < 0.0001). Both social and non-social feedback for wins were rated as positive (Turner syndrome, social condition: t = 5.35, *p* < 0.0001; Turner syndrome, non-social condition: t = 6.02, *p* < 0.0001; Control group, social condition: t = 7.05, *p* < 0.0001; Control group, non-social condition: t = 9.40, *p* < 0.0001). Taken together, this indicates that the points received during the task were perceived as valuable, and that the social and non-social feedback for wins induced positive affect.

**Table 2:** Affective ratings.

Affective rating	Turner syndrome	Control	Group comparison			
	M (SD)	M(SD)	*t*	*df*	*p*	*d*
Winning point (social)	4.84(1.29)	5.44(1.42)	− 1.82	65	.073	− 0.45
Winning point (non-social)	4.84(1.37)	5.42(1.02)	− 1.93	66	.058	− 0.48
Losing point (social)	3.52(1.06)	3.08(0.87)	1.80	65	.076	0.45
Losing point (non-social)	3.25(1.14)	2.91(1.12)	1.22	65	.228	0.30
Receiving social feedback	5.35(1.5)	5.47(1.25)	− 0.34	65	.731	− 0.09
Receiving non-social feedback	5.09(1.03)	5.64(1.05)	− 2.17	66	.034*	− 0.53

Participants rated their experiences of receiving feedback (smile or pile of money) and winning or losing a point. Note that the sample group size differs slightly in the different analyzes depending on the exclusion of incomplete data (see methods).

*p < .05; t = t-statistic; d = Cohen’s d.

As can be seen in Table 2, the control group rated the affective experience of receiving non-social feedback for correct choices significantly higher than the Turner syndrome group. No other group differences in affective experience ratings were found. In an explorative analysis, we added affective ratings of receiving feedback as a covariate in the main analysis. Since this did not change any of the results, this covariate was dropped from the final models.

### %Correct

**Figure 3:**
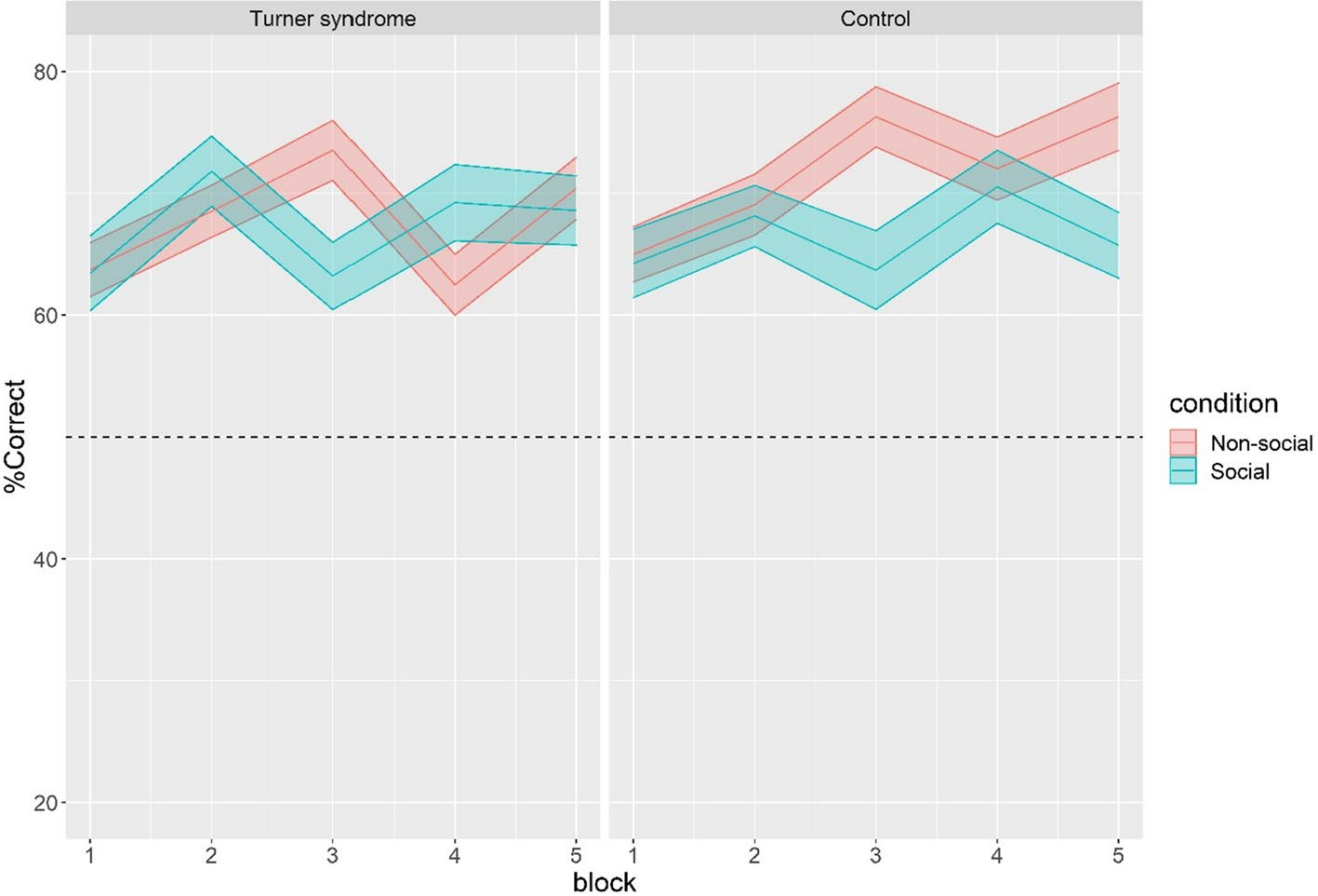
%Correct choices over the course of the experiment in participants with Turner syndrome and controls. The dashed line indicates chance level performance (50% correct choices). Trials were grouped into five blocks of 15 trials.

A significant main effect of block was found, reflecting higher %correct during later trials [b = 2.07, se = 0.65, *t* = 2.71, *p* = 0.007]. This demonstrates that, during the course of the experiment, there was a gradual increase in %correct choices, see Fig. 3. No significant main effects were found for condition [b = − 1.84, se = 2.76, *t* = − 0.86, *p* = 0.389], group [b = − 1.16, se = 3.73, *t* = − 0.43, *p* = 0.667], or order [b = 0.38, *se* = 2.87, *t* = 0.05, *p* = 0.961. There was also a significant group x condition interaction [b = 5.17, se = 2.23, *t* = 2.23, *p* = 0.026] but no significant interactions between group x block, group x order or condition x block (all *p* > 0.225).

**Figure 4:**
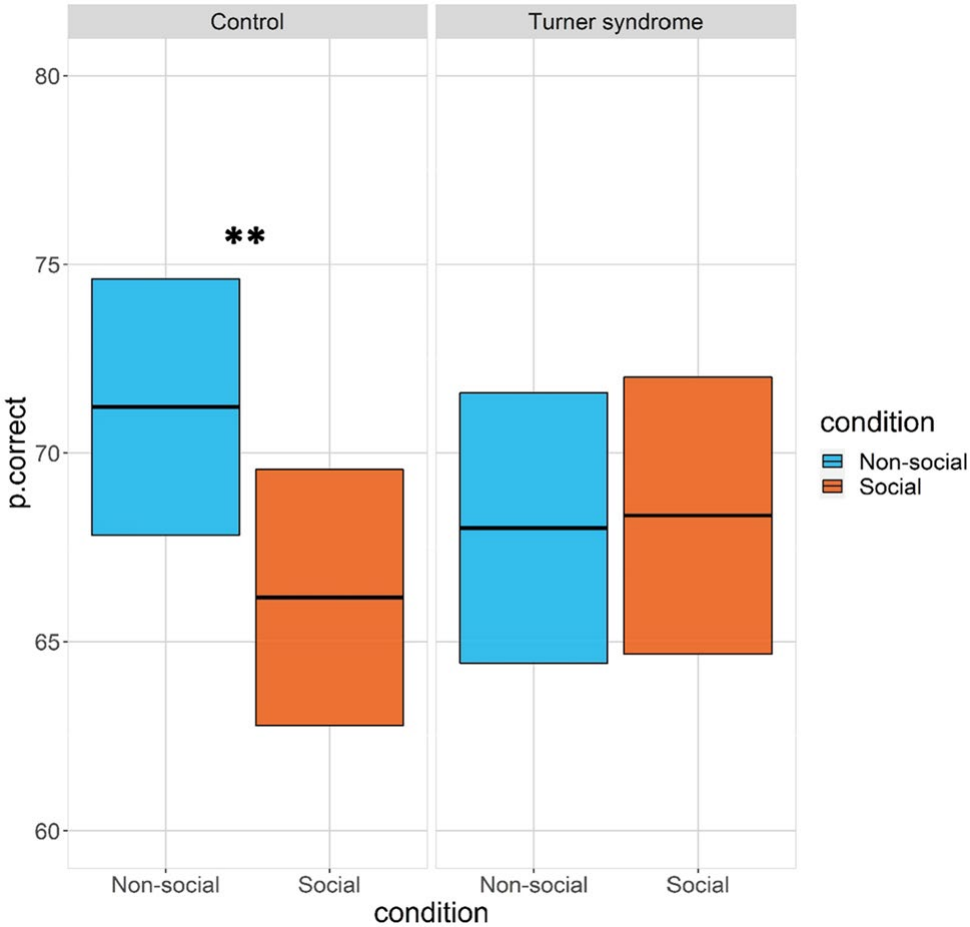
%Correct choices by group and condition. Pairwise follow-up tests: %correct choices by group and condition. Crossbars show estimated marginal means and 95% confidence intervals. *Note* ** *p* < .05 Bonferroni corrected.

Pairwise follow-up tests showed that the control group had a higher %correct in the non-social compared to the social condition [*b* = 5.01, *se* = 1.50, *t* = 3.45, *p* = 0.001] whereas no effect of condition was found in the Turner syndrome group [*b* = − 0.16, *se* = 1.64, *t* = 0.13, *p* = 0.898], see Fig. 4.

To examine the possibility that the development of %correct choices over the course of the experiment was best explained by a non-linear model, we ran the analysis again adding a quadratic term to the block variable. The model including the quadratic term did not have better fit than the linear model χ^2^ = 2.48, *p* = 0.479, suggesting no non-linear development.

### %Volatility

No significant main effects were found for condition [b = − 0.19, se = 2.94, *t* = − 0.08, *p* = 0.938], group [b = − 2.77, se = 4.72, *t* = − 0.37, *p* = 0.711], or order [b = 3,72, *se* = 4.11, *t* = 0.81, *p* = 0.419. The main effect of block was significant, indicating lower %volatility during later trials [b = 2.51, se = 0.69, *t* = 3.34, *p* = 0.001]. No significant interaction effects were found (all *p* > 0.242).

### %Switch-win

There was a non-significant trend towards lower %switch-win during later trials [main effect of block: b = − 1.74, se = 0.88, *t* = − 1.78, *p* = 0.074]. All other main and interaction effects were non-significant (all *p* > 0.20, all *t* < 1.27).

### %Switch-lose

No significant main effects were found for condition [b = − 10.11, se = 4.11, *t* = − 1.84, *p* = 0.065], group [b = 14.35, se = 6.35, *t* = 1.75, *p* = 0.082], or order [b = 3.02, *se* = 5.40, *t* = 0.51, *p* = 0.611. %Switch-lose was lower during later blocks [main effect of block: b = − 5.12, se = 0.97, *t* = − 5.35, *p* < 0.001]. Significant interaction effects were found between group and condition [b = − 7.57, se = 3.34, *t* = − 2.44, *p* = 0.015] and block and condition [b = 3.15, se = 1.15, *t* = − 2.55, *p* = 0.011] but not group x order [b = 2.92, se = 7.55, *t* = − 0.10, *p* = 0.917] or group x block [b = 0.26, se = 1.15, *t* = 0.62, *p* = 0.534].

Bonferroni corrected pairwise contrasts showed that, across blocks, the Turner syndrome group had a higher %switch-lose in the non-social as compared to the social condition [b = 8.26, se = 2.48, t = 2.80, *p* = 0.01] whereas no effect of condition on %switch-lose was found in the control group [b = 0.69, se = 2.25, t = 0.54, *p* > 0.90].

Bonferroni-corrected follow-up tests of the block by condition interaction showed that, across groups, %switch-lose decreased during the course of the task in both conditions, but to a higher degree in the non-social condition [b = − 4.99, se = 0.75, t = 6.60, *p* < 0.001] than the social condition [b = − 1.85, se = 0.74, t = 2.48, *p* = 0.036].

### Reinforcement learning parameters

#### α

Paired Wilcoxon tests showed no differences between *α* in the social and non-social conditions in the control group [social: Md = 0.61, MAD = 0.41; non-social: Md = 0.61, MAD = 0.37; V = 259, *p* = 0.369] or the group with Turner syndrome [social: Md = 0.63, MAD = 0.38; non-social: Md = 0.76, MAD = 0.28;V = 288, *p* = 0.053].

#### β

Across groups, *β* was higher in the non-social than in the social condition [main effect of condition: b = 0.54, se = 0.2, t = 2.89, *p* = 0.005]. No significant effects of group [b = 0.26, se = 0.28, t = 1.08, *p* = 0.281], or order [b = 0.17, *se* = 0.20, *t* = 0.72, *p* = 0.475] emerged. Older participants had higher *β,* reflected in a main effect of age [b = 0.02, *se* = 0.01, *t* = 2.57, *p* = 0.012]. The group by order interaction was not significant [b = 0.35, *se* = 0.29, *t* = 1.51, *p* = 0.136], but as hypothesized, a significant interaction between group and condition was found [b = 0.61, *se* = 0.29, *t* = 2.16, *p* = 0.035].

**Figure 5:**
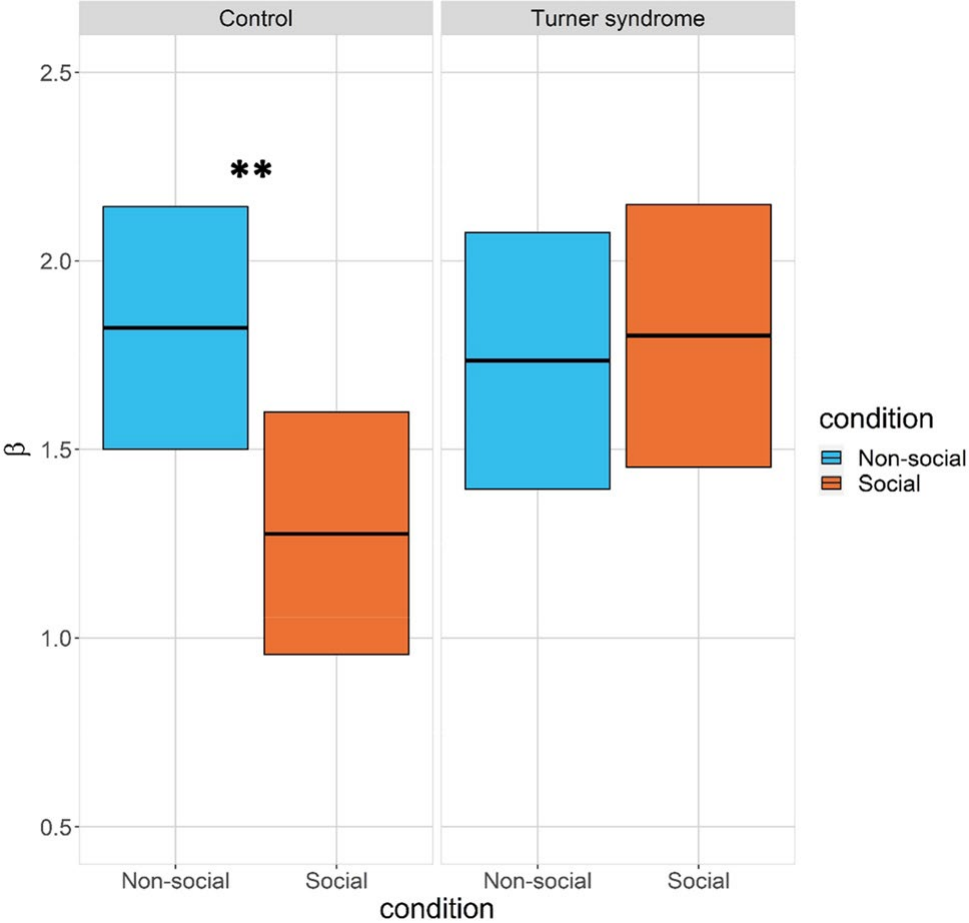
Bonferroni-corrected follow up comparisons. β in the control group and Turner syndrome group as a function of condition. Crossbars show estimated marginal means and 95% confidence intervals. *Note* ** *p* < .05 Bonferroni corrected.

Bonferroni-corrected follow up comparisons showed higher *β* in the control group in the non-social than in the social condition [b = 0.56, se = 0.20, t = 2.89, *p* = 0.01] whereas no effect of condition was found in the Turner syndrome group [b = − 0.07, se = 0.21, t = 0.27, *p* > 0.80], see Fig. 5*.*

## Discussion

Turner syndrome is associated with reduced social skills, an increased risk of having few friendship relations^12–15^, and difficulties with emotion recognition in faces^21,22^. The current study was the first to examine the effects of social feedback on reinforcement learning in Turner syndrome. As hypothesized, an attenuated effect of social feedback was seen in the group with Turner syndrome compared to the control group. Largely in line with previous literature^26,33,38^, social feedback caused a shift in the exploration–exploitation balance towards more exploration in the control group, but this effect was not found in Turner syndrome. Although social feedback affected reinforcement learning in controls by promoting a more explorative behavior, this effect was not beneficial in terms of task performance. Instead, social feedback reduced the proportion of correct choices relative to non-social feedback. Again, this effect was not seen in the Turner syndrome group. Instead, in this group, social feedback increased the probability to switch choice directly after loss but did not influence learning performance or reinforcement learning parameters.

Taken together, these results indicate a reduced influence of social feedback on reinforcement learning in Turner syndrome. Since reinforcement learning principles operate in many aspects of everyday learning and naturalistic behavior^59,60^, these alterations may be linked to altered social behavior. For example, an attenuated influence of social cues could potentially diminish the degree to which an individual adapts their behavior or beliefs to those of others.

An interesting finding was that for the control group, social cues were less effective than non-social cues in terms of overall task performance. This suggests that the shift towards exploration caused by social cues was not adaptive in the context of the current task. Social feedback may also have caused participants in the control group to attend to the potential mental or emotional states of the model rather than to the underlying probabilities inherent in the task. This could be detrimental for task performance, as the mental state of the model was not predictive of the true reward probabilities. This explanation would be consistent with previous studies showing that typically developed people tend to engage in spontaneous mentalizing in the presence of others^61^. The fact that social feedback increased reaction time variability suggests that this type of feedback resulted in additional cognitive processing than non-social feedback, possibly related to mentalizing.

As noted by Laland et al. (2004), social learning is likely to be adaptive in situations where social cues provide additional information than what can be acquired through trial and error^29^, something which was not the case in the current task. Importantly, social information effects on learning are often automatic and persist even if they are not adaptive^26,62^.

Interestingly, alterations in reinforcement learning have previously been described in ASD^63^, suggesting a potential overlap with Turner syndrome. Speculatively, reinforcement learning alterations may therefore be a shared mechanism underlying social challenges in Turner syndrome and ASD. Although it is not possible to determine the exact mechanisms underlying reduced performance after social feedback in the control group, the current study points to a previously not known area of social behavioral alterations in Turner syndrome. This suggests that reinforcement learning tasks may capture social cognitive challenges in Turner syndrome which are not readily assessed through self-reports. Interestingly, previous studies have suggested that self-ratings may underestimate social challenges in Turner syndrome^15,18,20^. As noted in the introduction, Turner syndrome is associated with executive function impairments^9–11^. Recent studies indicate that executive functions may interact with the core reinforcement learning mechanisms to shape learning^34^. For example, executive control of attention could affect which aspects of the stimuli that are attended to. An interesting possibility is therefore that the reduced effects of social feedback on reinforcement learning in Turner syndrome, seen in the current study, were modulated by executive function impairments. Although the current study suggests that women with Turner syndrome may be less influenced by social feedback during learning than controls, several similarities between participants with, and without Turner syndrome, should be noted. For example, both groups adhered to the general principles of reinforcement learning and neither group showed differences in learning rate (*β* parameter) between the social and non-social condition. Both groups showed similar reductions in reaction times and choice volatility over the course of the experiment, suggesting that attention impairments, which are common in Turner syndrome^10,11^ did not prevent participants from engaging in the task. It should be noted that some of these null findings may be an effect of the relatively small sample size.

## Conclusion, limitations, and suggestions for future research

Although statistical group differences were observed, it should be noted that the within-group variability was large. An interesting venue for future studies is to examine whether these individual differences are linked to genetic findings (e.g., karyotype or parental origin of the lost X chromosome) or psychiatric symptoms (e.g., symptoms of autism). Studies combining social reinforcement learning tasks with physiological measures such as pupillometry or fMRI are also likely to provide further insights into the underlying mechanisms of reinforcement learning alterations in Turner syndrome. A limitation of the current study is that no qualitative information about participants’ experiences, or interpretation of the task, was collected. This could have contributed further to our understanding of social reward learning in Turner syndrome.

Reinforcement learning tasks can be implemented in populations across wide age ranges and at different levels of cognitive ability. For example, a recent study using the same task, Kleberg et al. (2023) found that social cues *increased* the proportion of correct choices and increased sensitivity to rewards in people with Williams syndrome, whereas no effect of feedback type was seen in a group with intellectual disabilities of other genetic origins^44^. This suggests that social reinforcement learning alterations can provide insights into the phenotypical differences and overlap between genetic conditions (although it should be noted that Turner syndrome is rarely associated with intellectual disability).

Online data collection is a feasible way to collect data and to include more participants, even those who do not live near research facilities. This may be particularly important in research on rare genetic disorders, where large sample sizes are difficult to achieve. However, there are limitations with this method, as we were not able to directly observe participants during the task or monitor the environment where they performed it. Furthermore, as data collection took place online, we were not able to complete a more thorough psychiatric or medical assessment, including medication history. Futures studies should ideally compare data collected online and in a research environment.

In this study, we used social and non-social feedback on the correct choice when performing a probabilistic learning task. An interesting future study would be to compare the effects of social and non-social feedback for both wins and losses. A social stimulus in the form of a face with a negative emotional expression in case of loss/wrong response would be particularly interesting to investigate in individuals with Turner syndrome, since they have shown particular difficulties in reading faces that convey anger or fear^21–23^*.* Despite these limitations, this is, to our knowledge, the first study to show that women with Turner syndrome may be less sensitive to social influences in probabilistic learning than the general population.

### Supplementary Information

Supplementary Information.

## Data Availability

The data that support the findings of this study are available from the corresponding author, H.B.A., upon reasonable request.
