# Supplementary material for: Reduced effects of social feedback on learning in Turner syndrome
Source: Sci Rep. 2023 Sep 22;13:15858. doi: 10.1038/s41598-023-42628-7 (PMC10516979; doi:10.1038/s41598-023-42628-7)
Supplement: Supplementary file 1 — Supplementary Information. [file 41598_2023_42628_MOESM1_ESM.docx]

Supplement

***Computational modeling***

This study is methodologically conducted in a similar way to our previous study "Social feedback enhances learning in Williams syndrome" by Kleberg et al. (2023)^1^ and the content of this supplement may therefore overlap to some extent.

*Parameter estimation*

Data were modeled at the level of the individual. Model comparison and parameter estimation were performed through maximum likelihood estimation using the MATLAB function *fminbnd*. As in previous publications ^2,3^, data were analyzed in two stages. First, the parameter values which maximized the log likelihood estimate (LLE) of the observed data were selected. Secondly, we restrained using the parameter values using Gaussian priors generated in stage one. Specifically, the log likelihood of the parameters under the prior distribution were added to the LLE of the observed parameters, a procedure shown to increase model fit and reduce the risk of extreme parameter values^4^.

Following previous studies^5^, we computed individual Aikaike weights for the candidate models based on the Aikaike information criterion (AIC). The AIC introduces a punishment on the LLE for models with a higher number of free parameters^6^. For model *i* with *V* number of free parameters, AIC is:

-2* log(L_i_) + 2*V (*Eq. 1)*

Here, L_i_ is the maximum likelihood of the model. AIC values were transformed to Aikaike weights following^6^. These range from 0-1 and reflect the relative performance of each considered model relative to the others. To avoid local minima, maximum likelihood estimation was repeated 50 times for each individual and condition using randomly generated starting points and the values resulting in the highest likelihood were selected.

*Candidate models*

Following a previous study using the same task, we considered the following candidate models:

*Model 1.* The standard Rescorla-Wagner model, described in the main text, has the free parameters α and β. For details, see *Computational modeling* in the main manuscript. The fit of this model was compared to alternative reinforcement learning models (models 2 - 4) and alternative models (models 5 - 6) described below.

*Model 2* includes separate learning rates (α) for positive and negative outcomes but is otherwise identical to model 1.

*Model 3* updates both the value of the chosen option (V_c_) and the non-chosen option (V_nc_) but is otherwise identical to model 1. Specifically, the value of the non-chosen action (nc) is updated according to:

$V{(nc)}_{t+1}={V(nc)}_{\begin{aligned} t \\ \end{aligned}}+ \alpha(1-r_{t}-{V\left( c \right)}_{t})$ *(Eq.2)*

*Model 4* is an extension of model 3 which includes a loss/reward weight parameter which governs the relative weight given to losses and rewards^3,7^. Here, the outcome value *r* is determined by the free parameter *d* in the range [0,1]*,* so that:

r = 1-d *(Eq.3)*

if the trial resulted in a win, and

r = -d *(Eq.4)*

if the trial resulted in a loss. If d = 0.5, both outcomes are given equal weight. As in model 3, values of both the chosen and the unchosen options are updated.

*Model 5* assumes that participants switch between the two choice alternatives at each trial with a probability of 1, regardless of observed outcomes. This model was included to control for the possibility that participants merely switched between alternatives without considering the outcomes.

*Model 6* assumes that participants choose at random, meaning that the probability of each choice is 0.5 throughout the experiment, regardless of observed outcomes. This model was included to control for the possibility that participants responded by random rather than being influenced by outcomes.

*Model fit*

AIC weights of the candidate models are shown in *Figure S1.* As can be seen, model 1 had best fit in the control group in both conditions and in the Turner syndrome group in the social condition, although the difference from model 4 was small. Model 4 had best fit in the Turner syndrome group in the non-social condition. We modeled the data using model 1 in both groups and conditions to facilitate comparison.


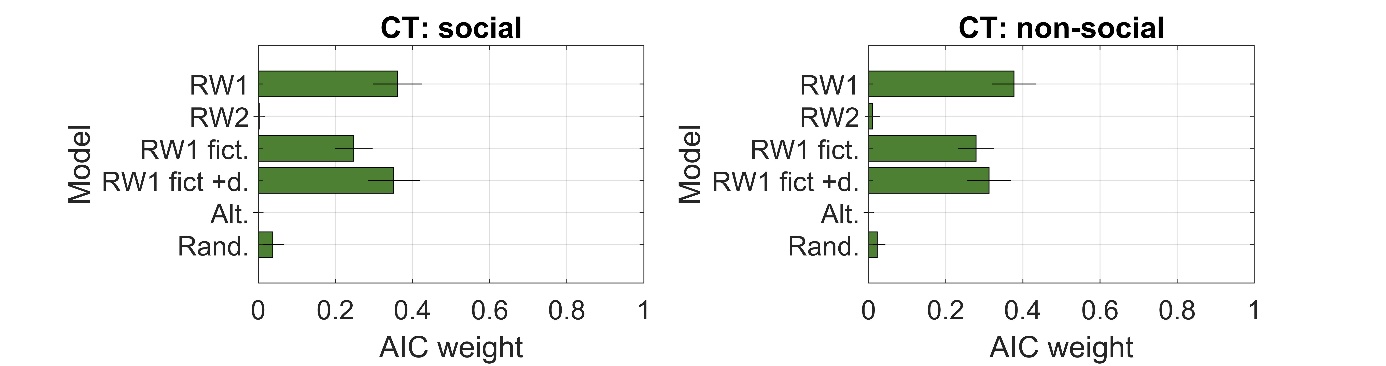

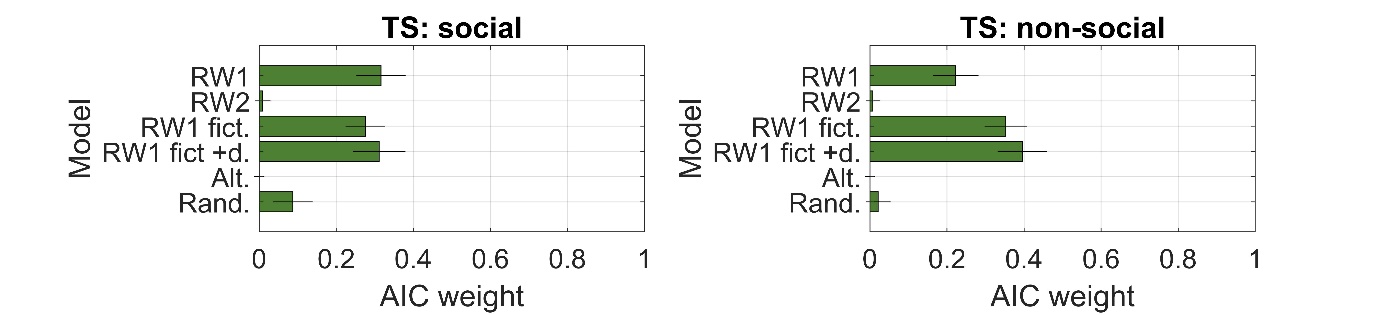


*Figure S1.* AIC weights for candidate models by group and condition. CT = Control group; TS = Turner syndrome.

*Parameter Recovery*

We simulated data from 300 agents with α and β values in the range observed in the actual data and analyzed these data using the methods described under *Parameter estimation.* Correlations between recovered and simulated values were r = .80 for both α and β, indicating adequate parameter recovery.

*Model Validation*

We simulated 150 data sets for each participant in which the simulated agent completed the task with the same parameter values as the actual participant. These data were then compared to the actual observed data. Figure S2 shows average %correct choices in the observed data (black lines) plotted against a colored area covering the mean and 95% confidence interval of the simulated data for each group and condition. As can be seen, the simulated data appears to capture the dynamics of observed choice behavior.


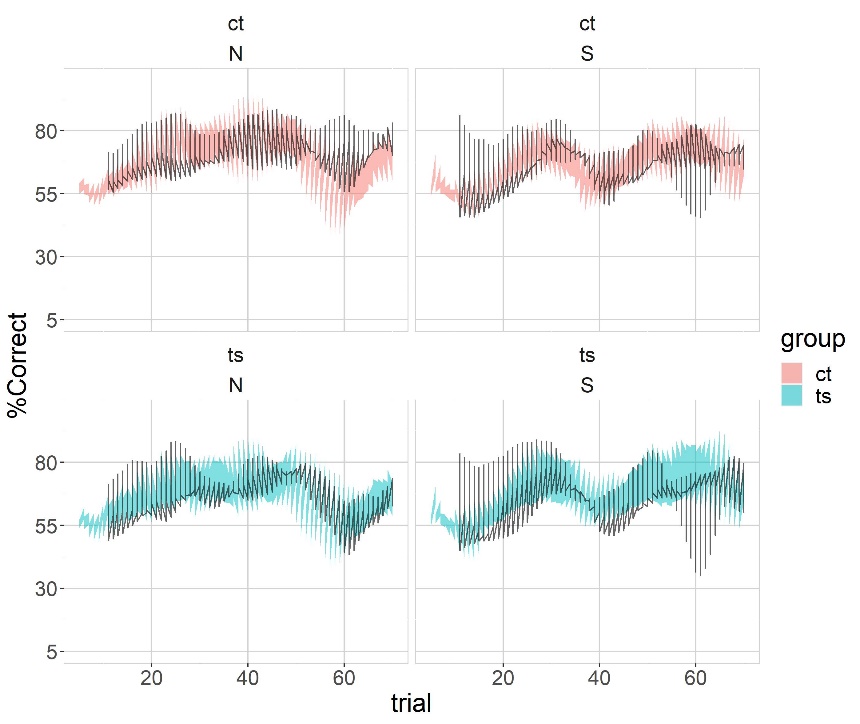


*Figure S2.* Observed %correct (black lines) and predicted %correct based on simulation (colored area) by group and condition.

***References***

1 Kleberg, J. L. *et al.* Social feedback enhances learning in Williams syndrome. *Scientific Reports* **13**, 1-11 (2023).

2 Den Ouden, H. E. *et al.* Dissociable effects of dopamine and serotonin on reversal learning. *Neuron* **80**, 1090-1100 (2013).

3 Frey, A.-L., Frank, M. J. & McCabe, C. Social reinforcement learning as a predictor of real-life experiences in individuals with high and low depressive symptomatology. *Psychological medicine* **51**, 408-415 (2021).

4 Daw, N. D. Trial-by-trial data analysis using computational models. *Decision making, affect, and learning: Attention and performance XXIII* **23** (2011).

5 Stengård, E. & Van den Berg, R. Imperfect Bayesian inference in visual perception. *PLoS computational biology* **15**, e1006465 (2019).

6 Wagenmakers, E.-J. & Farrell, S. AIC model selection using Akaike weights. *Psychonomic bulletin & review* **11**, 192-196 (2004).

7 Gold, J. M. *et al.* Negative symptoms and the failure to represent the expected reward value of actions: behavioral and computational modeling evidence. *Archives of general psychiatry* **69**, 129-138 (2012).
